# Mouldable Conductive Plastic with Optimised Mechanical Properties

**DOI:** 10.3390/polym16030311

**Published:** 2024-01-23

**Authors:** Arfat Anis, Abdullah Alhamidi, Zahir Bashir, Mohammad Asif Alam, Saeed M. Al-Zahrani

**Affiliations:** 1 SABIC Polymer Research Center (SPRC), Chemical Engineering Department, King Saud University, Riyadh 11421, Saudi Arabia; akfhk90@hotmail.com (A.A.); szahrani@ksu.edu.sa (S.M.A.-Z.); 2 Catenated Carbon Consultancy Ltd., 192 Wake Green Road, Birmingham B13 9QE, UK; zbashir2703@gmail.com; 3 Center of Excellence for Research in Engineering Materials (CEREM), King Saud University, Riyadh 11421, Saudi Arabia; moalam@ksu.edu.sa

**Keywords:** reinforced polymer composite, polymer blend, recycled PET, carbon fibre, conductive plastic, mechanical properties

## Abstract

This paper investigates making an injection mouldable conductive plastic formulation that aims for conductivity into the electromagnetic interference (EMI) shielding range, with good mechanical properties (i.e., stiffness, strength, and impact resistance). While conductivity in the range (electrostatic charge dissipation) and EMI shielding have been attained by incorporating conductive fillers such as carbon black, metals powders, and new materials, such as carbon nanotubes (CNTs), this often occurs with a drop in tensile strength, elongation-to-break resistance, and impact resistance. It is most often the case that the incorporation of high modulus fillers leads to an increase in modulus but a drop in strength and impact resistance. In this work, we have used short carbon fibres as the conductive filler and selected a 50/50 PBT/rPET (recycled PET) for the plastic matrix. Carbon fibres are cheaper than CNTs and graphenes. The PBT/rPET has low melt viscosity and crystallises sufficiently fast during injection moulding. To improve impact resistance, a styrene-ethylene-butadiene-styrene (SEBS) rubber toughening agent was added to the plastic. The PBT/rPET had very low-impact resistance and the SEBS provided rubber toughening to it; however, the rubber caused a drop in the tensile modulus and strength. The short carbon fibre restored the modulus and strength, which reached higher value than the PBT/rPET while providing the conductivity. Scanning electron microscope pictures showed quite good bonding of the current filler (CF) to the PBT/rPET. An injection mouldable conductive plastic with high conductivity and raised modulus, strength, and impact resistance could be made.

## 1. Introduction

Intrinsically conducting polymers where the chains conduct electricity are few and are based on π-conjugated chains. Shirakawa et al., started the trend with the discovery of polyacetylene [1]. The conductivity of polyacetylene doped with iodine could reach 10^5^ S/m, which is metal like conductivity. Polyaniline, polythiophene, and polypyrrole are of the same type of π-conjugated system [2,3]. Such polymers are not injection mouldable due to lack of fusability, hence their applications have gravitated to organo-electronic devices such as light-emitting diodes, biosensors, water purification devices, and hydrogen storage.

Mouldable conductive plastics with a semi-conductor range of electrical conductivity have found greater use. Conductivity is achieved by filling an insulating common plastic with intrinsically conductive fillers. Examples of conductive fillers are carbon black, metal powders, carbon fibres, carbon nanotubes and graphene, and metal-coated carbon fibres. Semi-conductor level of conductivity can be achieved. There are three levels of electrical conductivity in conductive plastics: (1) anti-static, (2) electrostatic dissipation (ESD), and (3) electromagnetic dissipation.

Conductive plastics would prevent the build-up of high amounts of charge as they would allow it to leak away more gently. With a conductive plastic, if earthed, a static charge build-up can be reliably prevented.

In electronic circuitry, which is adjacent or near to other electrical and electronic devices, electromagnetic interference (EMI) is a problem as it can distort signals and create noise. EM waves should not penetrate into the boxes housing the circuits as it would cause interference in electronic circuitry. Metal boxes provide the ultimate shielding but the weight is a penalty, and the transport sector requires to reduce weight for minimizing fuel consumption. However, metal boxes, if not earthed properly, can cause electric shocks from loosened wires. If the electronics are boxed in a standard plastic box, radio frequency (RF) interference from neighbouring circuits is possible. Conventional methods for achieving EMI shielding with plastic enclosures employ a metal coating by deposition, plating, stamping, and so on. Drawbacks are besides the expense, scratches and delamination of the conductive coating leads to performance failures. Alternatively, a conductive plastic can give a box that has good performance for EMI shielding, and it is much lighter and cheaper than an aluminium box. Further, it offers higher design flexibility.

The academic studies on conductive plastics have unearthed methods to reach percolation at lower loadings of filler particles. The percolation is defined as the filler content where there is massive rise in conductivity, and it is correlated to the condition where filler particles are touching and show connectivity. One concept is the segregated network. In this, the filler is not distributed uniformly in the polymer matrix but is segregated and concentrated in certain areas, leading to connectivity. An example is metal particles mixed with PVC powder and compression moulded [4,5]. The metal powder sits on the surface of the PVC powder. A sheet is formed, with the metal particles following the contours of the PVC particles, leading to connectivity. However, compression moulding is limited to forming sheet shapes and it is a slow, batch process, hence it is seldom used industrially. Although mechanical properties are not reported in such studies [4], it is almost certain that segregating fillers to polymer particle boundaries would lead to a weak and brittle material. One may be able to have samples to measure conductivity, but it would be unusable practically.

A second approach is to use an immiscible polymer blend instead of a single polymer as the matrix to achieve the segregated network, through the concept of ‘double percolation’ [6,7]. Depending on thermodynamic properties, the filler might (1) distribute evenly in the two phases, in which case there is no advantage over a single polymer; (2) have preference to be one polymer phase than the other; or (3) it may segregate and concentrate in the regions between the two-phase boundaries leading to connectivity at low concentrations.

The order of mixing of the binary polymer blend can be used to control the spatial location of the filler [8,9]. The filler is incorporated by melt mixing in a first polymer with which it has less affinity and is pelletised. Next, the pellets of the first polymer containing the filler is melt mixed with the second polymer. The ratios of the two polymers is chosen to give a co-continuous morphology. The filler that was in the first polymer with low affinity then migrates to the second polymer’s domains. Depending on the mixing time, the migration process can be arrested when the filler is at the boundaries of the two polymer phases, thereby creating a segregated network. The carbon fillers (carbon black, carbon fibres, CNTs, and graphene) have shown tendency for preferential localization in polymer blends compared with metal fillers. However, the question mark with the segregated network in blends is weak interfaces and compromised mechanical properties but this is often uninvestigated.

For a conductive plastic to be useful, conductivity is a necessary but not sufficient condition. There are practical difficulties in making the articles, without compromising the mechanical properties severely. With high conductivity plastics for injection moulding, one difficulty in processing may be the high melt viscosity. In the finished article, often the problem is brittleness and poor impact properties.

In this work, we have chosen short carbon fibres (CF) as the conductive filler. While CF does not have the highest intrinsic conductivity, it is higher than carbon black and has lower density than metals. We have avoided carbon nano tubes and graphene due to their expense and the agglomeration tendency of the nano materials. PBT-rPET was chosen as the matrix, not so much to obtain a co-continuous matrix but because rPET gives a low melt viscosity and good surface finish, while the PBT crystallises fast and induces the slow-crystallising rPET to crystallise as well. The PBT-rPET system allows ease of injection moulding and dimensional stability of the moulded article when used at a higher temperature than the Tg of the constituent polymers. In addition, to counter the tendency for a drop in impact properties, a third polymer, namely a styrene-ethylene-butadiene-styrene (SEBS) rubber, was added. A low T_g_ material provides rubber toughening but with an inevitable drop in modulus and strength. However, the CF has a high modulus and high strength; hence, we thought it could offset the drop from the rubber. The electrical conductivity sought is preferably in the EMI range.

## 2. Materials and Methods

### 2.1. Materials

A 50:50 by weight of a PBT/rPET blend was prepared and used for this study. PBT is poly(butylene terephthalate) and rPET is recycled poly(ethylene terephthalate. The PBT resin, grade PBT-R1-G0-011, was provided by Sipchem Chemical Company, Al Khobar, Saudi Arabia. The recycled PET polymer resin had an I.V. of 0.7 dL/g and was made by extruding flakes and pelletising. Afterwards, the PBT and rPET pellets were compounded to make a 50:50 PBT/rPET blend.

Carbon fibre was chosen as the conductive filler. Polyacrylonitrile—based chopped carbon fibres of 6 mm length (Trade name: Tenax^®^ HT C493) were obtained from Toho Tenax GmbH, Germany. The fibres were supplied as bundles of about 6 mm length.

The bundled fibres were held together with a protective polyurethane size and could not be melt compounded well with the 50:50 PBT/rPET blend due to poor dispersion. Hence, the bundles were desized with acetone. However, the desized fibres were too fluffy and could not be fed to the mini twin screw extruder. Hence, the bundles were put in a bladed mixer to unloosen the fibres. It was then found that the mechanically debundled carbon fibres could be fed to the extruder for compounding with the 50:50 PBT/rPET blend with good dispersion. Mechanical debundling is an important step in making the conductive plastic using this carbon fibre.

Kraton, a SEBS rubber was used as an impact modifier (33.33 wt.% Kraton FG1901 GT and 66.67 wt.% Kraton G1657 VS). This was obtained as white pellets from Kraton Polymers LLC, Belpre, OH, USA.

### 2.2. Preparation of Composites

Various quantities of CF were mixed with the polyester blend. The PBT/rPET/CF composites were prepared using the melt compounding method, followed by injection moulding to produce specimens for subsequent characterizations. To prevent hydrolysis during the melt mixing process, the pellets of the pre-compounded PBT/rPET blend underwent drying in an oven at 120 °C for 24 h before compounding with CF. The pre-compounded dried PBT/rPET pellets, Kraton rubber pellets, and CF were manually mixed and then fed simultaneously into the compounder. The blending of CF composites was conducted using a benchtop 15 cc micro-compounder (DSM Xplore, Sittard, The Netherlands) equipped with co-rotating twin screws. The melt mixing duration was limited to 3 min to prevent transesterification reactions, and the temperature was set to 260 °C with a mixing speed of 100 rpm. The extruded melt was collected in a collector attachment with a cylindrical piston, which could be connected to the DSM Xplore injection moulding machine (a microinjection moulder with a capacity of 12 cm^3^). This setup allowed the production of specimens with various shapes for tensile testing (ASTM D638 [10]), flexure, and Izod impact testing (ASTM D790 [11] and ASTM D256 [12], respectively), as well as electrical and thermal conductivity measurements. Table 1 provides detailed information on the composition of all the fabricated composite specimens.

### 2.3. Characterization of the Composites

#### 2.3.1. Scanning Electron Microscope (SEM)

The morphology of carbon fibres post-coating with Au through sputtering was examined using the SEM 1 (Model JSM-6360A, JEOL Ltd., Tokyo, Japan) at an accelerating voltage of 15 kV. Additionally, the cross-sectional morphology of the cryo-fractured PBT/rPET/CF composites was investigated using another SEM 2. Gold coating was applied to the samples using a Quorum Q150R S sputter coater (Quorum Technologies Ltd., Laughton, UK) at 20 mA for 90 s. Visualization was conducted with a ZEISS EVO LS 10 SEM (Carl Zeiss Microscopy GmbH, Jena, Germany) at an accelerating voltage of 20.0 kV. Image capture was facilitated by the ZEISS SmartSEM version 5.05 software (Roduit, N., Geneva, Switzerland).

#### 2.3.2. Differential Scanning Calorimetry (DSC)

DSC was applied to investigate the melting and crystallisation characteristics of both the pure 50/50 PBT/rPET blend and the 50/50 PBT/rPET/CF composites. The analysis was conducted using a DSC-60A instrument from Shimadzu, Tokyo, Japan. The specimens underwent heating from 30 to 280 °C at a rate of 10 °C/min. Following this, they were maintained at 280 °C for 3 min to erase any thermal history before being cooled to 30 °C at a rate of 10 °C/min.

#### 2.3.3. Notched Izod Impact Test

To evaluate the toughness of the PBT/rPET/CF composites, the notched Izod impact test was performed using an AMSE pendulum impact tester from Torino, Italy, following ASTM D 256 [12] standards. The moulded bar adhered to the specified dimensions of a length of 64 mm, width of 12.7 mm, and thickness of 3.25 mm as outlined in the standard. The samples were notched at the midpoint of the bar, 31.8 mm from one end, with a depth of 2.5 mm. Positioned vertically, the notched side faced towards the pendulum, and fracture occurred through the application of a hammer with an impact velocity of 3.50 m/s and an energy of 5.5 J. To ensure repeatability ten samples of each composite composition were tested and the average values along with standard deviations were reported.

#### 2.3.4. Tensile Testing

Tensile testing was conducted utilizing a universal testing machine (specifically, the H100KS model of the uniaxial universal testing machine located in Horsham, PA, USA). The testing parameters were set with a test speed of 5 mm/min and a gauge length of 50 mm. Standard test bars, having dimensions of 150 mm in length, 12.70 mm in width, and 3.25 mm in thickness, were employed. The reported values are the averages of measurements on five test samples encompassing tensile force, tensile strength, tensile modulus, strain at maximum, and strain at break (elongation).

#### 2.3.5. Flexural Testing

The flexural properties, including flexural strength, flexural modulus, and strain at maximum stress, were assessed using a Hounsfield H100KS Series testing machine equipped with a 3-point bending loading system. The support span length was fixed at 52 mm, and the crosshead speed was maintained at 2 mm/min in accordance with ASTM D 790-03 [11] standards. The standard test bars had dimensions of 134 mm in length, 12.7 mm in width, and 3.25 mm in thickness. To ensure repeatability five samples of each composition were tested and the averages values along with standard deviations were reported.

#### 2.3.6. Thermal Conductivity

The thermal conductivity of both the pure blend and its composites was gauged using a thermal conductivity analyser from C-Therm Technologies Ltd. (Fredericton, NB, Canada) in accordance with ASTM D7984 [13] standards. The instrument utilizes a Modified Transient Plane Source Sensor for thermal conductivity measurement. The reported values were average of three measurements conducted at ambient room temperature (25 °C). The samples tested were 2 mm in thickness.

#### 2.3.7. Electrical Resistivity Measurement

Resistivity measurements were conducted on injection-moulded plaques of dimensions of 40 mm in length, 40 mm in width, and 2 mm in thickness. ASTM D-257 [14] standards were employed for testing the resistive samples, utilizing a Keithley Electrometer/High Resistance Meter (Model: 6517B) along with a Keithley Model 8009 Resistivity Chamber. For the conductive samples, testing followed the International Electrotechnical Commission (IEC 60093) method, using a low-resistivity meter (Loresta-GP, Mitsubishi, Tokyo, Japan) in conjunction with an ESP-type four-probe (Model MCP-TP08P). The reported values represent the averages obtained from measurements on three samples conducted at ambient room temperature (25 °C).

#### 2.3.8. Shrinkage Measurements

The change in dimensions of the as-moulded bars for the neat blend without Kraton, the blend with Kraton, and their respective composites with CF was evaluated following annealing at 170 °C in an oven for a duration of 1 h. Flexural bars were uniformly cut to a length of 60 mm, and the percentage of shrinkage was subsequently calculated. The experiments were conducted twice, and measurements of the length after heat exposure were recorded.

## 3. Results and Discussions

**Figure 1:**
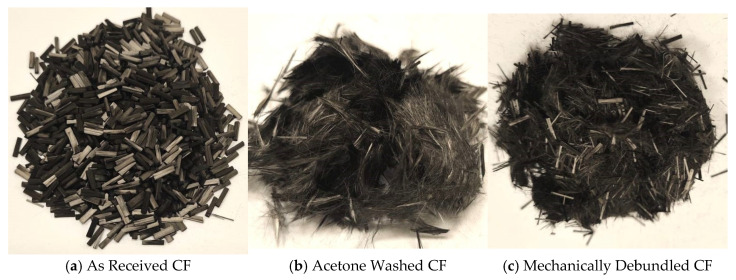
Carbon fibre, as received and after debundling. The acetone washed CF bundles were too fluffy and unusable, but the mechanically debundled CF was feedable to the extruder for melt compounding with the polyester blend.

**Figure 2:**
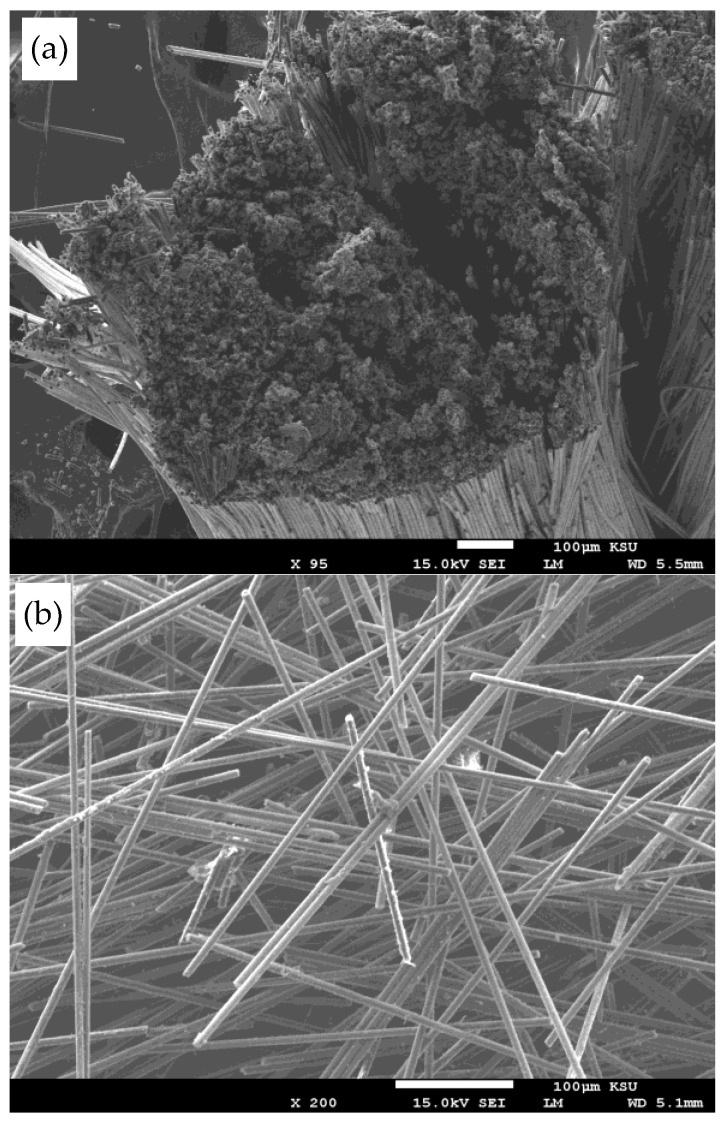
(**a**) SEM photograph of the carbon fibre bundle (HTC 493, 6 mm) before debundling. (**b**) SEM photograph of a carbon fibres (HTC 493, 6 mm) after mechanical debundling.

The chopped carbon fibres were received as 6 mm long bundles, held together with a polyurethane size. A crucial step for melt compounding the CF with the polyester blend was to unbundle them. Figure 1a shows the bundles of chopped carbon fibre, as received. The as-received bundles showed poor dispersion when melt compounded with the polyester blend. Hence, the bundles were de-sized by immersing in acetone. On drying, this yielded a CF mass that was too fluffy to feed to the extruder for melt compounding with the polyester blend (Figure 1b). Hence, a mechanical debundling procedure was attempted. The mechanical debundling opened up the fibres (Figure 1c) sufficiently and they could be fed to the extruder, with uniform dispersion and distribution. There were some intact bundles left (Figure 1c). Of course, mechanical debundling might break some of the fibres and reduce the aspect ratio. However, this debundling procedure was essential and it managed to get both the desired rise in electrical conductivity and mechanical properties. Figure 2 shows the SEM images of the fibre bundles as received and after mechanical debundling.

### 3.1. Electrical and Thermal Conductivity

**Figure 3:**
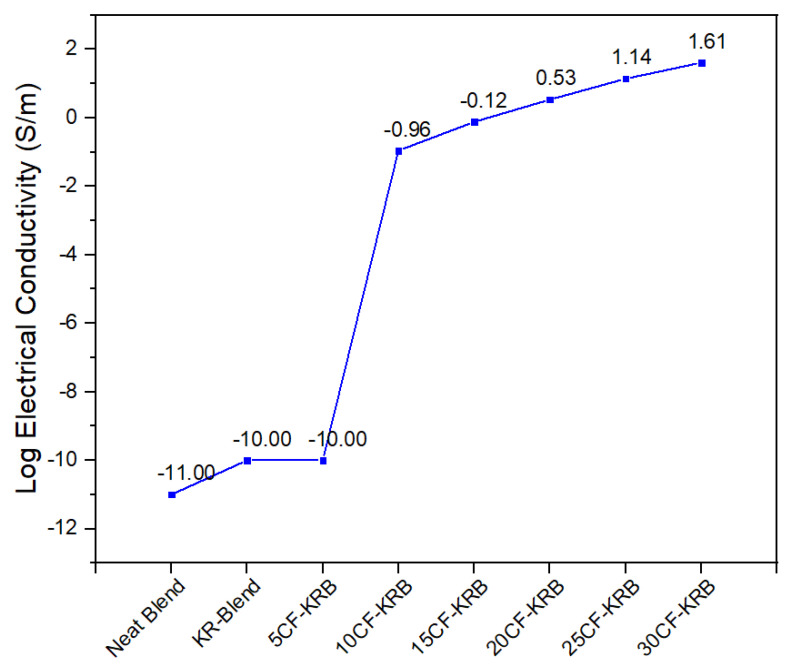
Electrical conductivity of neat blend (50:50 PBT-rPET), KR-Blend (50:50 PBT-rPET Blend (80 wt.%) + Kraton rubber (20 wt.%)), and KR-Blend with CF. The percolation threshold is between 5 and 10% of CF.

Electrical resistivity was actually measured, but the conductivity is its inverse. Figure 3 shows the electrical conductivity of PBT-rPET-Kraton rubber filled with chopped carbon fibres. The percolation threshold was between 5–10% loading. Both the PBT-rPET and PBT-rPET-Kraton are insulators with a conductivity of 10^−11^ to 10^−10^ S/m. Adding 10% CF raised the conductivity to 0.110 S/m. Thereafter, the conductivity increased with CF content but at a slower rate. At 20%, the conductivity was 3.414 S/m, at 25% it was 13.760 S/m and with 30% CF, the value reached 40.933 S/m. To get a perspective, metals have a conductivity of 1.45 × 10^6^ S/m (steel) to 6.30 × 10^7^ (silver); semi-conductor germanium has a conductivity of 2.17 S/m and silicon has a value of 1.56 × 10^−3^ S/m.

Electrically conductive plastics have a conductivity of 10^−10^ to 10^−6^ S/m for ESD (electrostatic discharge) applications, 10^−6^ S/m to 1 S/m for partly conductive usages, and above 1 S/m for EMI shielding usages. The conductive plastic made here with 20–30% CF reached values >1 S/m. That is, they reached the range for the EMI application, while the values for 10 and 20% CF were well above that needed for ESD.

CF has a lower electrical conductivity of 10^6^ S/m while bulk Al has a conductivity 3.538 × 10^7^ S/m; however, we found previously [15] that Al-PET composites were insulators even with 25% of irregularly shaped Al particles. In PBT-PET with 30% nano platelet Al, it achieved only an ESD level of conductivity (1.4 × 10^−6^ S/m). The Al in PET and PBT-PET did not degrade the mechanical properties and in fact had even improved it. The appearance and gloss of Al platelets filled PBT-PET blends was very good due to a glossy metallic effect. However, the conductivity performance was lower than one would expect from the conductivity of bulk aluminium. This was perhaps due to the shape of the particle (irregular, platelet, nano spherical) or because Al had a 6 nm thick insulating oxide coating, and a 6 nm thick oxide coating is a higher proportion in nano Al platelet compared with a 6 nm coating on bulk aluminium, hence the oxide reduces the conductivity when the Al is in nano form. Due to this, we opted for CF as the filler in this work.

**Figure 4:**
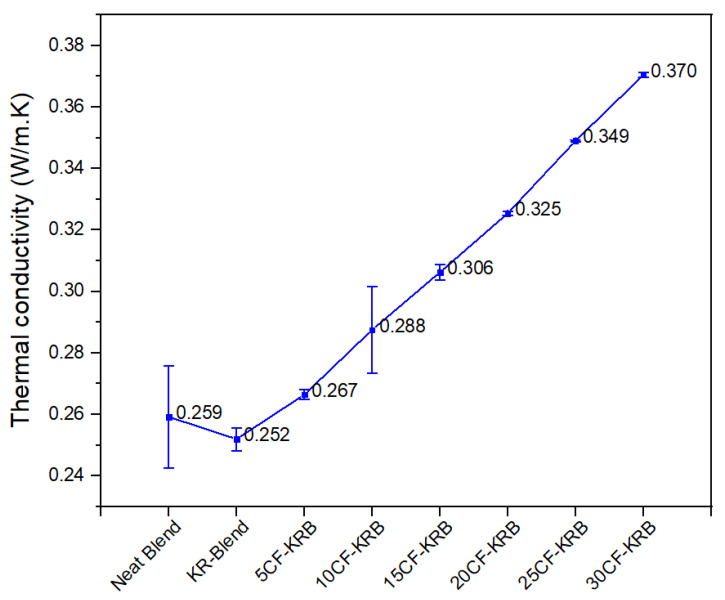
Thermal conductivity of neat blend (50:50 PBT-rPET), KR-Blend (50:50 PBT-rPET Blend (80 wt.%) + Kraton rubber (20 wt.%)), and KR-blend with CF.

Figure 4 shows the thermal conductivity of the polyester blend with CF. There is a modest rise in thermal conductivity. Unlike electrical conductivity, percolation is generally not achieved with thermal conductivity till about 60% or 70% filler content by volume. Further, the rise is not by several orders of magnitude unlike with electrical conductivity. The rise in thermal conductivity with CF in PBT-rPET-Kraton blend is about half that achieved with PBT-rPET blend filled with nano Al platelets. To be considered a thermally conductive plastic, the thermal conductivity must be over 1 W/m.K. The CF filled PBT-rPET-Kraton blend can be considered to be in the category ‘electrically conductive but thermally insulative’.

### 3.2. Crystallisation of Components of the Blend

A PBT-PET blend consists of two crystallisable polymers of which PBT crystallises fast and PET too slowly for injection moulding. Previous work with PBT-PET by AlHamidi et al. [15]) has shown that 60:40 PBT-PET melts are transparent (suggestive of a single phase in the melt), and when the melt is cooled, intriguingly there is only one exothermic peak in the DSC. This could lead to the misleading indication of crystallisation of both in a single phase. Aramova et al. [16] demonstrated how quench cooling can lead to an amorphous PBT-PET blend. However, PBT-PET melts are not miscible; the two polymers crystallise separately. Moreover, the PBT speeds up the crystallisation of the PET [17].

**Figure 5:**
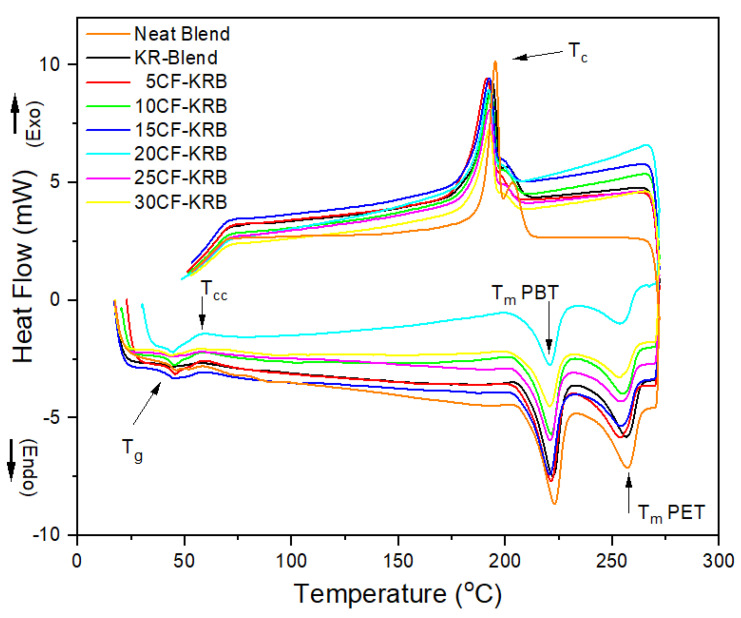
DSC heating and cooling curves of neat blend (50:50 PBT-rPET), KR-Blend (50:50 PBT-rPET Blend (80 wt.%) + Kraton rubber (20 wt.%)), and KR-blend with CF.

The DSC heating curve of a bar of the 50:50 PBT-rPET in Figure 5 are similar to the ones in the literature of PBT-PET [15,18]. Figure 5 shows a T_g_ just below 50 °C followed by a cold crystallisation exotherm immediately after (see arrow T_cc_). Our previous work with injection moulding of PBT and 60/40 PBT/PET blends had shown that there is a thin, amorphous, transparent skin jacketing the bar, which is visible to the naked eye. The same transparent skin was seen on this 50/50 PBT/rPET. The exotherm in Figure 5 (see arrow T_cc_) is due to the cold crystallisation of the amorphous skin and possibly further crystallisation of the slow component, the PET domains.

In the heating curve, after cold crystallisation, there are two endothermic peaks, the first corresponding to PBT domains and the second to the PET domains. In other words, the PBT and PET crystallise in separate domains.

In the heating curve for the PBT-rPET-Kraton (unfilled and CF filled), the Kraton is a SBS block copolymer. The rubbery domains have a T_g_ of −50 °C which is below the scanning range. The styrenic block should have a T_g_ around 100 °C, but this was too weak to be seen in the heating curve of Figure 5.

In Figure 5, on cooling from the melt, there are also two (partially overlapping) exothermic crystallisation peaks. Previous works [15,18] showed a single crystallisation peak, but here there is evidence that one of the two polymers crystallises first followed by the second. This is possibly because the rPET had a lower molecular weight.

### 3.3. Shape Stability at High Temperature

**Figure 6:**
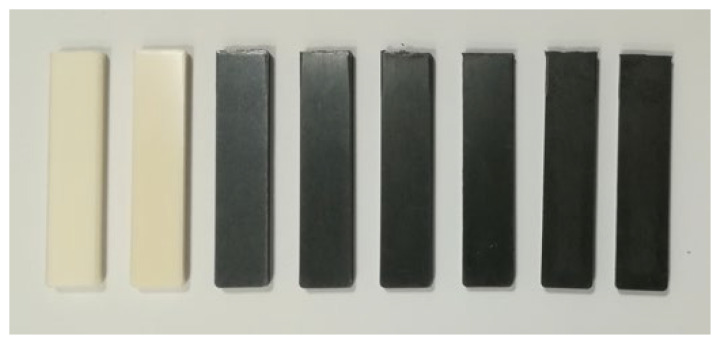
From the left: White bar of neat blend (50/50 PBT/rPET); white bar of KR-Blend (50/50 PBT/rPET with 20 wt.% Kraton SEBS rubber toughener; black bars with 5, 10, 15, 20, 25, and 30% CF in KR-Blend.

Figure 6 shows the appearance of the specimen bars after heating to 170 °C. The first bar on the left shows the 50/50 PBT/rPET; it is opaque. If only PET was used then the bar would have been transparent and amorphous as PET is a slow crystalliser. PBT is a fast crystalliser, but its melt viscosity is higher than PET. A 50/50 PBT/rPET is a polyester composition that can be used for injection moulding where a combination of lower viscosity and good surface finish comes from the PET and the fast crystallisation in the time scale of injection moulding comes from PBT. If crystallisation is complete in the as-moulded article, the part would have dimensional stability if heated above the T_g_.

The second bar from the left in Figure 6 shows the PBT/rPET with the toughening SEBS rubber Kraton added to it. Visually, there is not much difference apart from a slight yellowing, but there are changes on addition of Kraton in mechanical properties, which will be discussed.

The remaining bars in Figure 6 show the addition of carbon fibres to the PBT/rPET with Kraton gives a mouldable composition even with 30% CF. However, even 5% CF gives a black product. Thus, the use of CF as the conductive filler limits the article to one colour. Our previous work showed if Al flakes are used as the conductive filler, a silvery metallic appearance is obtained, but if spherical Al powder is used, a dark grey appearance is obtained [19].

One concern is the dimensional stability versus service temperature when using the moulded article above the T_g_ of the two polymers. If amorphous PET is used, it will cold crystallise above its T_g_ (78 °C), and there would be massive shrinkage and shape distortion [15]. Table 2 shows the change in the length of moulded bars after annealing at 170 °C for 1 h. The PBT/rPET bar shrank by 1.8%, but after adding SEBS it was somewhat lower at 1.3%. This shrinkage is due to the crystallisation of the thin amorphous skin of the bar. The addition of CF immobilises the matrix so that the shrinkage goes down to zero.

**Figure 7:**
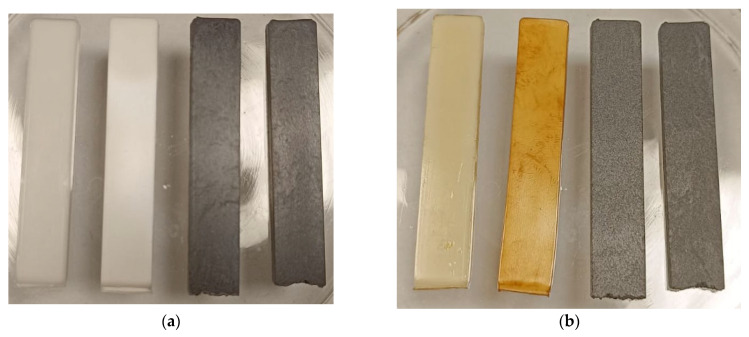
(**a**): Bars before heating (Neat Blend, Blend with Kraton, 15% CF KR-Blend and 30% CF KR-Blend; (**b**): After heating to 230 °C for 1 h.

Figure 7 shows the effect of heating the bars to 230 °C for 1 h. This is a temperature in between the two melting peaks of PBT and PET (see DSC in Figure 5). At this temperature, the bar with Kraton shows discolouration due to degradation of the Kraton. The bars surprisingly did not lose their shape although the PBT (half the material) is molten. However, the bars showed sticking to the glass (because the PBT component had melted), but the flow and sticking was much less with the bars filled with CF. Thus, short time exposure of CF filled 50:50 PBT/rPET to even 230 °C is possible.

### 3.4. Mechanical Properties

Having established sound mouldability, shape stability, and electrical conductivity up to EMI shielding levels, the next important aspect was whether the mechanical properties were intact or degraded (as is often the case).

**Figure 8:**
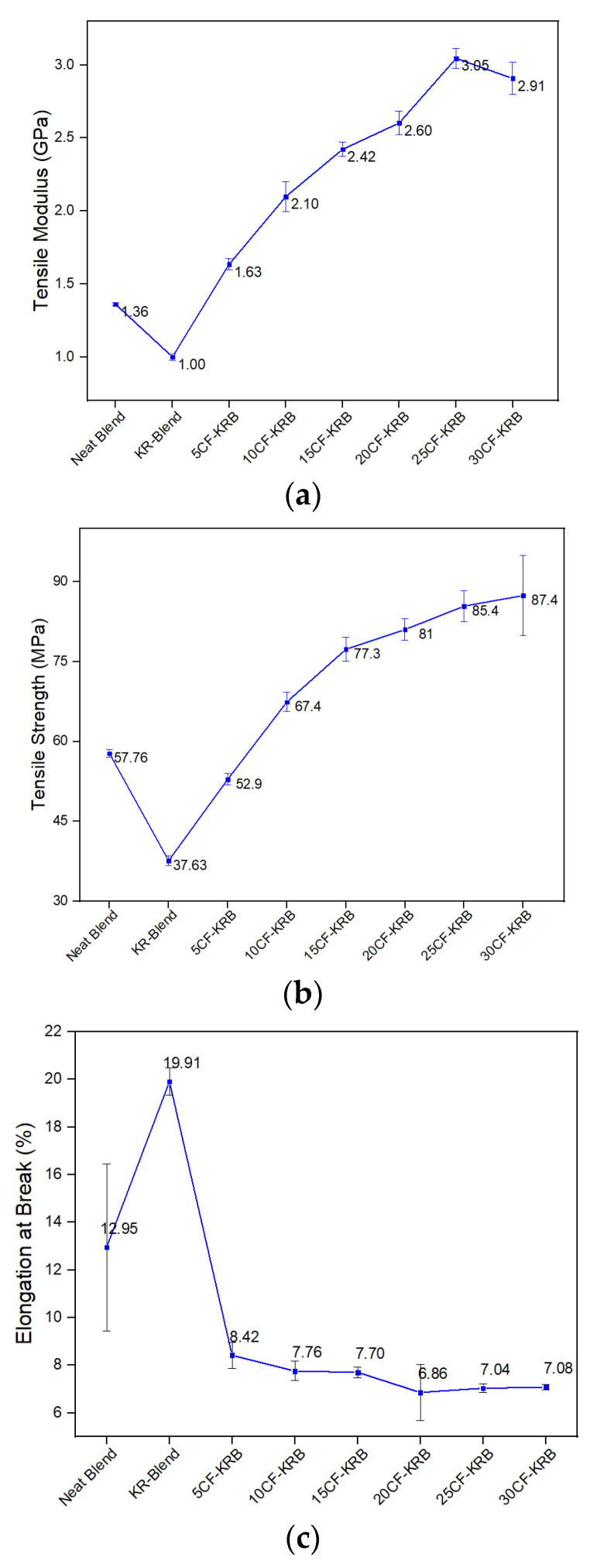
(**a**) Tensile modulus values of neat blend (50:50 PBT-rPET), KR-Blend (50:50 PBT-rPET Blend (80 wt.%) + Kraton rubber (20 wt.%)), and KR-Blend with CF. (**b**) Tensile strength values of the same. (**c**) Elongation-at-break values of the same.

Figure 8a shows the tensile modulus of the 50/50 PBT/rPET blend was 1.36 GPa. Adding 20% of SEBS Kraton rubber caused a drop in modulus to 1.00 GPa, as expected. However, with the addition of CF, the modulus increased and more than compensated the drop from the rubber. No doubt the orientation tendency of the CF parallel to the flow, especially at higher fibre contents such as 20% and above, might contribute partially to the increased value [20].

Figure 8b shows the strength also follows a similar profile—a drop after addition of the Kraton, followed by an increase when CF is present. The tensile strength of the polyester blend was 57.8 MPa, which dropped to 37.3 MPa when 20% Kraton was present, but increased to 87.4 MPa (i.e., a value higher than the blend on its own). The rise in strength shows the CF has a reinforcement effect. The value will also reflect an orientation effect (i.e., a higher axial strength). The transverse strength could not be measured. In many cases, the addition of particulate fillers causes a drop in strength [21].

Figure 8c shows the elongation-to-break. The addition of the Kraton SBS rubber caused a spike in the elongation. Adding CF caused the customary decrease in elongation seen when rigid fillers are added. However, the elongation-to-break with even 30% CF was 7.1%, whereas it would have dropped to under 2% or 1% if the rubber was not present, which would have made the material brittle.

**Figure 9:**
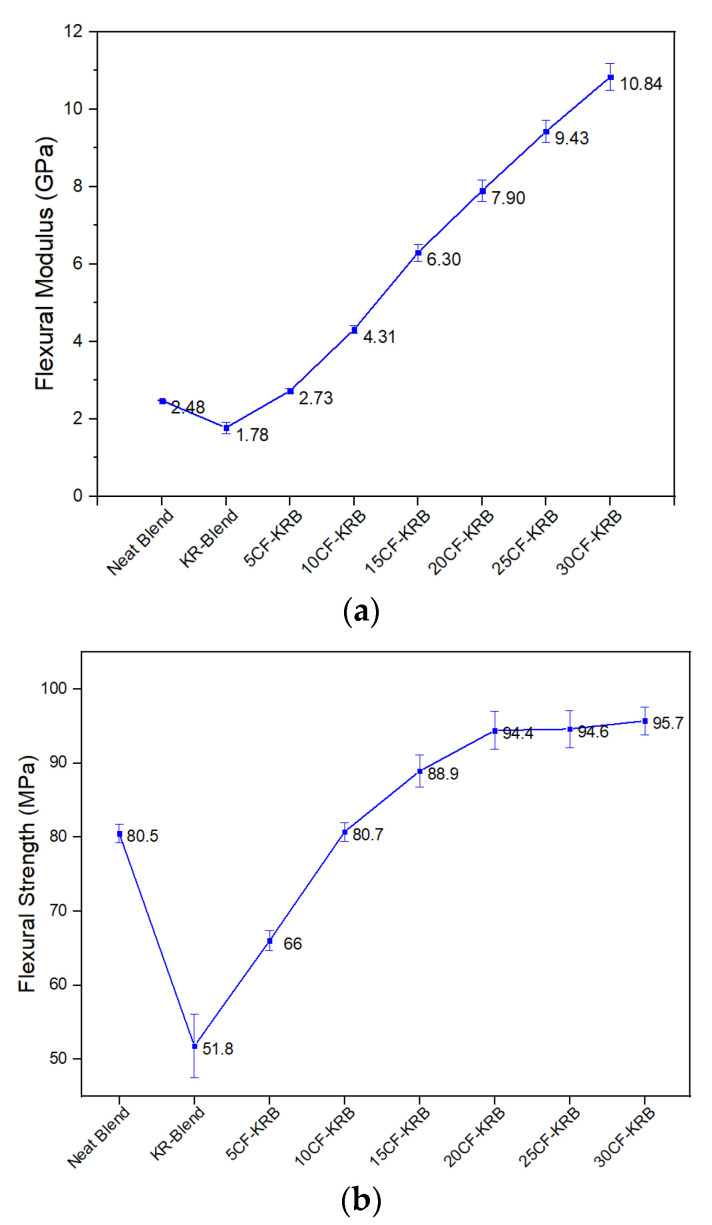
(**a**) Flexural modulus values of neat blend (50:50 PBT-rPET), KR-Blend (50:50 PBT-rPET Blend (80 wt.%) + Kraton rubber (20 wt.%)), and KR-Blend with CF. (**b**) Flexural strength values of the same.

Figure 9a,b show the flexural modulus and strength mirror the tensile modulus and strength. There is a drop in flexural modulus and strength on addition of Kraton, but this is reversed on addition of CF.

**Figure 10:**
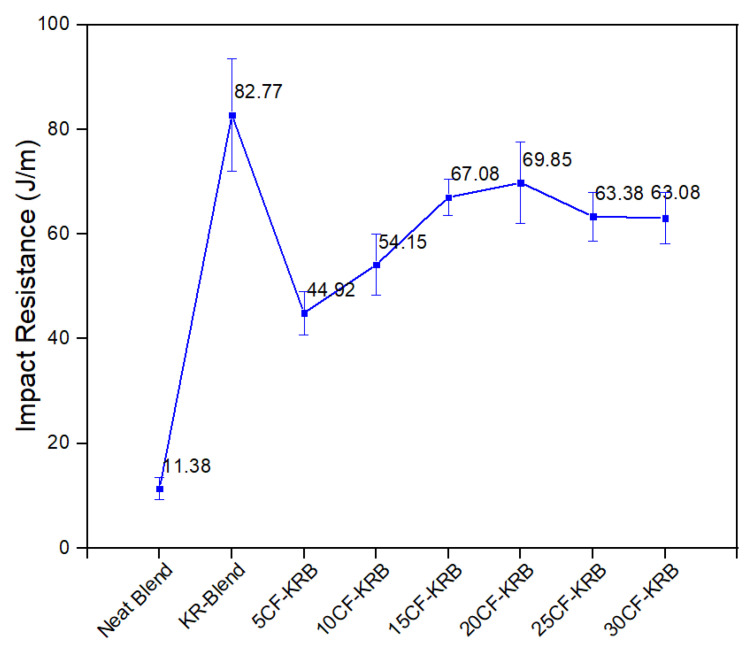
Notched Izod impact resistance of neat blend (50:50 PBT-rPET), KR-Blend (50:50 PBT-rPET Blend (80 wt.%) + Kraton rubber (20 wt.%)), and KR-Blend with CF.

Figure 10 shows the notched Izod impact resistance. This shows a spike in the opposite direction on addition of Kraton rubber to the polyester blend. The impact resistance of the 50/50 PBT/rPET we used in this work was very much lower at 11.4 J/m than a 60/40 PBT/PET blend we made previously by compounding the PBT and PET (33 J/m). This polyester blend is quite brittle and comparable to atactic polystyrene, which means dropping an article made from it on to a hard surface would cause breakage. However, on addition of the Kraton to the blend, the impact resistance increased to 83.7 J/m. This clearly shows the rubber particles were effective in impact toughening. On the addition of the CF, the impact resistance decreased but it was still well above the value of the blend, at around 63.38 J/m with 30% CF. A conductive composite with an Izod impact over 100 J/m would be ideal.

A rubber-toughened plastic does not automatically translate that toughening property when fibres are added to such a plastic. McGrath [22] notes that attempts at improving the toughness of composites by increasing the toughness of the plastic matrix have given disappointing results in that a large increase in matrix toughness has not translated into a similar increase in composite toughness.

Nevertheless, we now have a mechanically sound enough platform for a conductive plastic with high electrical conductivity but with good modulus, strength, and reasonable impact resistance.

The mechanical and electrical properties obtained with CF in 50:50 PBT-rPET may be contrasted with works using CF with PET or PBT only as the matrix. The drawback of using PET is its slow crystallisation, which is sometimes not recognised by those interested in making composites with PET as matrix. With a PET matrix, moulded articles would be amorphous near the surface and semi-crystalline in the core. If the moulded CF-PET article is used above the T_g_ (78 °C for PET), the CF would restrain the high shrinkage found with unfilled PET, but there would still be higher shrinkage compared with a PBT or PBT/PET matrix. This effect would be amplified because the thermal conductivity is raised when CF is added (see Figure 4) and the heat would be conducted to the mould faster increasing the quenching effect on the PET melt.

Shi et al. [23] studied the effect of surface treatment of CF on the mechanical properties of CF/PET composites. The tensile strength increased linearly with CF content going from 54 MPa for PET to 80 MPa with 15% CF. However, the impact increased and reached a maximum at only 6% CF and then decreased to a value below PET by 15% CF. By treating the CF with nitric acid and applying different sizings, Shi et al.’s [23] impact strength increased by approximately 10%. In our work, despite the presence of Kraton rubber, the tensile strength at 15% CF had a similar value (77 MPa) as Shi et al., (80 MPa), while the impact resistance increased by about 6× the value of the PBT/rPET matrix even at 30% CF [23].

PBT is usually preferred in place of PET for injection moulding (while PET is used for oriented fibres) because of its faster crystallisation. Karsli et al. [24] studied the effect of the CF sizing on the mechanical properties. They compounded PBT granules and 30 wt.% of 6 mm chopped carbon fibres (similar to our CF). With their best size (polyurethane), they got a modulus of 5.5 GPa for the 30% CF-PBT; we got about 3 GPa at 30% CF/PBT-rPET (ours is lowered due to the Kraton rubber toughener). Their strength with 30% CF/PBT was 120 MPa −130 MPa with the best size (polyurethane). We got about 80 MPa for the strength; the lower value was due to the addition of Kraton. 80 MPa is still higher than the strength of unfilled plastics. Karsli et al. [24], did not report the impact resistance of their 30% CF-PBT. They got an electrical conductivity of about 10^−2^ S/cm for the 30% weight CF-PBT composites with various sizings. We obtained higher electrical conductivities—10% CF, a conductivity of 0.110 S/m was obtained, and with 30% CF, the value reached 40.933 S/m.

Hwang [25] measured the tensile properties, electrical conductivity, and EMI shielding efficiency of solid and foamed CF/PBT composite. An electrical conductivity of 2.5 × 10^−2^ S/m was obtained with the solid material. The tensile strength values increased and reached a maximum at 8 wt.% fibre content but levelled off at 13 wt.%. The decrease in the tensile strength was attributed to the aggregation of fibres. The maximum CF loading was 13 wt.% which caused reduction of the tensile strength, but this loading was found to be not good enough for the EMI shielding effect. To have a good EMI Shielding Effectiveness value, Hwang stated the CF loading must be greater than 30 wt.%. However, he did not show the tensile strength at 30% CF, but if aggregation took place at 13%, the strength would have decreased greatly at 30% CF. The impact properties were not shown, but can be expected to be impaired if aggregation took place at 13% CF. It is thus likely at the 30% CF content which showed EMI shielding, there was a drastic drop in mechanical properties. Here, we think our procedure for the debundling of the 6 mm CF was effective. Debundling by acetone washing was inappropriate as it not only made the fibres too fluffy to feed (Figure 1 and Figure 2); it washed away the polyurethane sizing that Karsli et al. [24] showed was the best size for PBT matrix. The mechanical debundling used here before compounding with 50/50 PBT/rPET reduced the aggregation of the CF which Hwang faced, while preserving the polyurethane size.

Kim et al. [26] made conductive polymers using a PBT blend with poly(acrylonitrile-co-styrene-co-acrylate) and a hybrid filler of CF and CNTs. The composite with CF and PBT- PolyASA gave a conductivity of 9 S/m, while the one with CF and CNT gave a higher electrical conductivity (1.4 × 10^2^ S/m). PolyASA consists of 60 wt.% of poly(butyl acrylate) as a rubbery phase and 40% of poly(styrene-co-acrylonitrile) (SAN). Hence, this paper sought to optimise the electrical conductivity with CF and the expensive CNTs, and the mechanical and flame-retardant properties with a co-polymer with rubbery phase. The impact was considered. The value for the PBT was not given (from the literature, the notched Izod impact is 7.8 kJ/m^2^ for PBT or about 52 J/m). The impact resistance of the CF- PBT- PolyASA was 5.4 KJ m^−2^ (that is, it is lower than the PBT); for the CF-CNT- PBT- PolyASA, the impact was 6.4 KJ m^−2^, which is lower than that of the PBT. That is, the conductive composition of CF- PBT- PolyASA had a lower impact resistance than the PBT. Hence, they attained a higher conductivity than us due to the use of CNTs in addition to the CF, but at the expense of impact resistance which was reduced to below that of PBT.

### 3.5. Morphology

It is well known that a 50:50 blend of two immiscible polymers leads to a co-continuous morphology [27]. The two phases are intertwined. In a previous paper on Al flakes in 60/40 PBT/PET composites, we showed that 60/40 PBT/PET blends do form a co-continuous morphology [16]. However, unlike most immiscible polymer blends where the intertwined domains are microns in size, in PBT/PET it is sub-micron or nm in size, and this nano size and the low contrast between PBT and PET made imaging them very difficult. While the co-continuous morphology has been used as a tool to make conductive polymers with a low percolation threshold by selectively concentrating the conductive filler either in one phase, or at the interface of the domains, it appears to us now that this would not be feasible with the 50/50 or 60/40 PBT/PET blend because the CF of diameter 7 μm and length up to 6 mm cannot be concentrated in one or the other matrix whose domains are sub-micron. In this work, the only rationale for using 50:50 PBT/rPET is because it gives a high melting engineering plastic with easy mouldability (low viscosity and good gloss), and capability for use at temperatures beyond 170 °C. In the SEM pictures that follow, it is impossible to identify the PBT/PET co-continuous morphology, due to its small size, and the presence of the other fillers (i.e., Kraton and CF). Hence, the PBT/PET co-continuous morphology should be treated in the current case like a single phase in which Kraton particles and CF are embedded. We shall examine the distribution of the latter two and their bonding, and orientation, and see if they are in line with the mechanical properties.

Rubber toughening of plastics is an important area of study especially for the use of engineering plastics. Such plastics have high modulus and high strength, and high temperature and chemical resistance, but are let down by low elongation-to-break and low-impact resistance. Rubber toughening is frequently needed for engineering plastics. In the principles of rubber toughening, the type of rubber, good dispersion, optimum particle size (>100 nm but <5 μm), inter particle distance, and bondability are considered as important factors.

**Figure 11:**
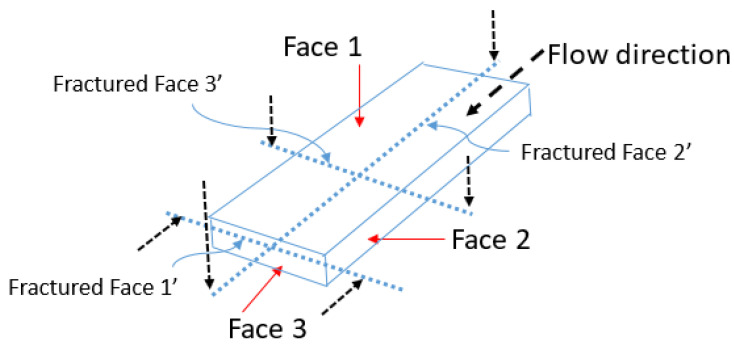
SEM images were based on (1) a cryo-fractured surface in the middle of the bar parallel to Face 3 and (2) a face parallel to Face 2 and (3) a face parallel to Face 1, fractured along the blue dotted line axis, the black dashed lines show the direction of propagation of the fracture.

**Figure 12:**
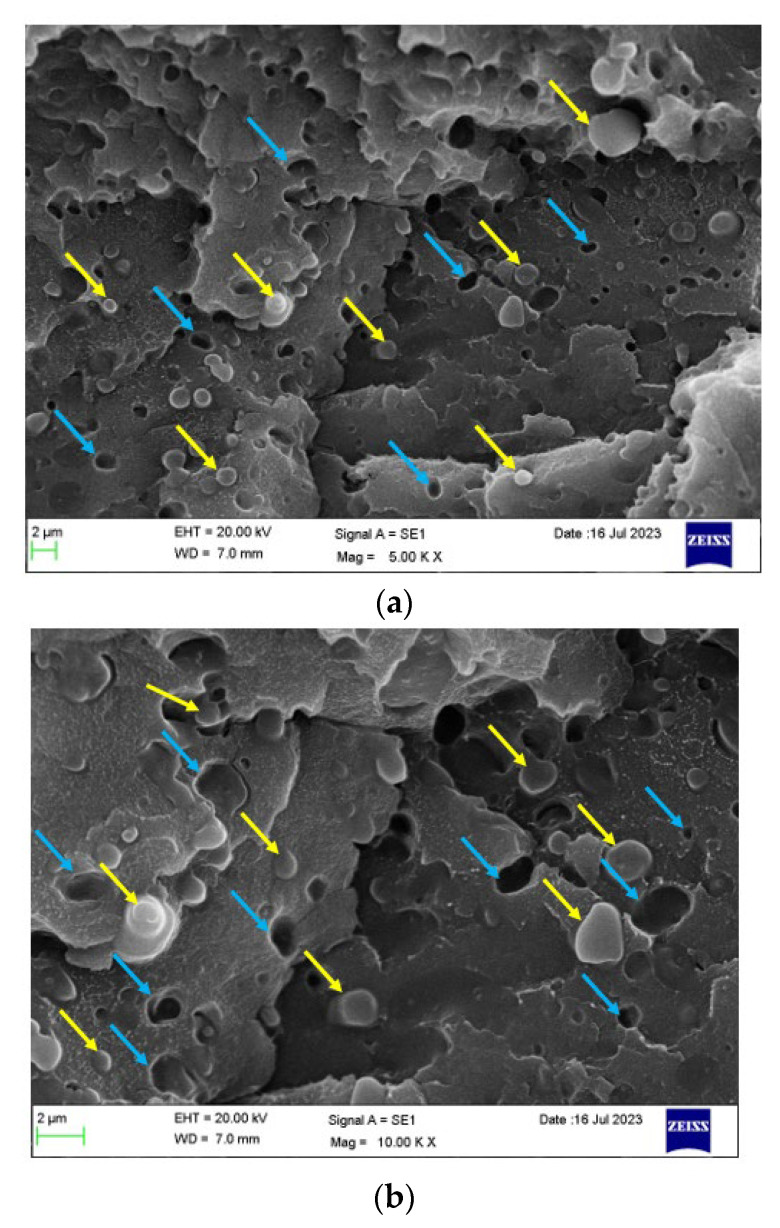
(**a**) Cryo-fractured cross section (Face 3’) of a bar of PBT/rPET (50/50)/Kraton (80/20) blend. Yellow arrows show Kraton particles, and the blue arrows show the drop outs due to Kraton pull out. (**b**) Higher magnification, cryo-fractured cross section surface (Face 3’) of a bar of PBT/rPET (50/50)/Kraton (80/20) blend. Yellow arrows show embedded and well adhered Kraton particles, and the blue arrows show the drop outs due to Kraton pull out.

**Figure 13:**
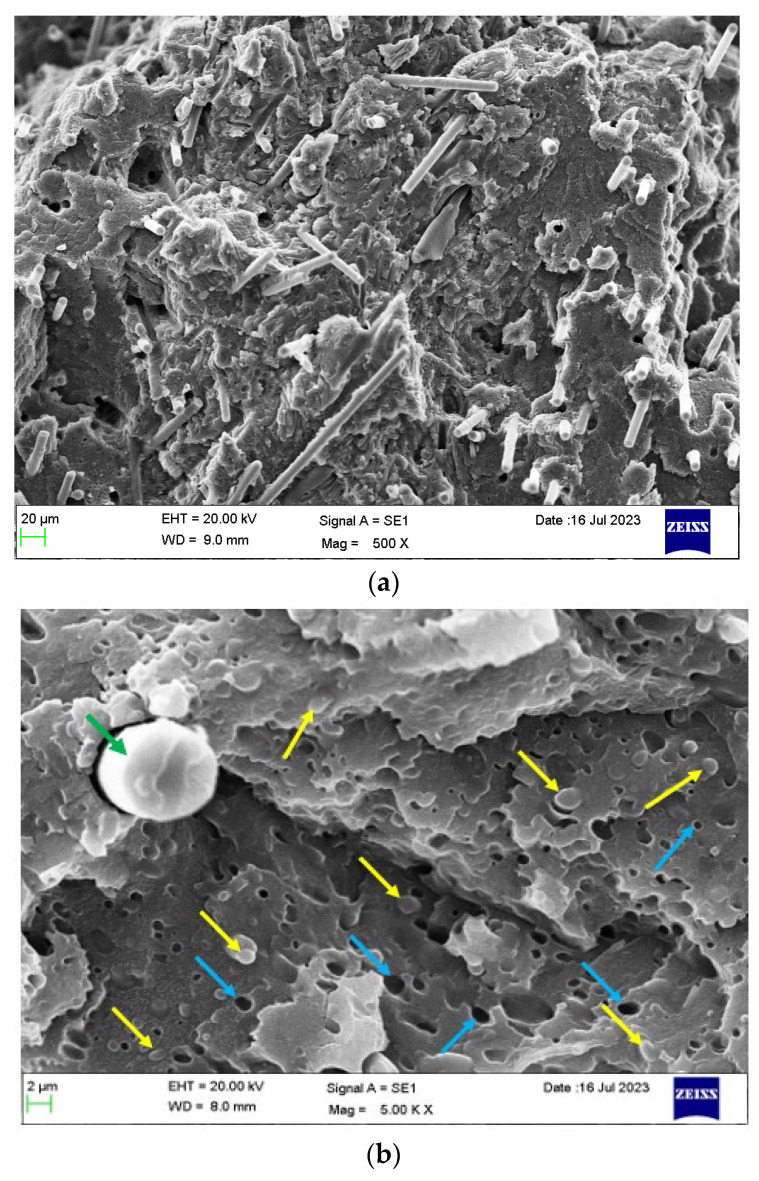
(**a**) Cryo-fractured cross section (Face 3’) of a bar of PBT/rPET (50/50)/Kraton (80/20) blend, with 5% CF. (**b**)**.** Higher magnification cryo-fractured cross section (Face 3’) of the same surface Green arrow shows a partially debonded CF, yellow arrows show Kraton particles, and the blue arrows show the drop outs due to Kraton pull out.

**Figure 14:**
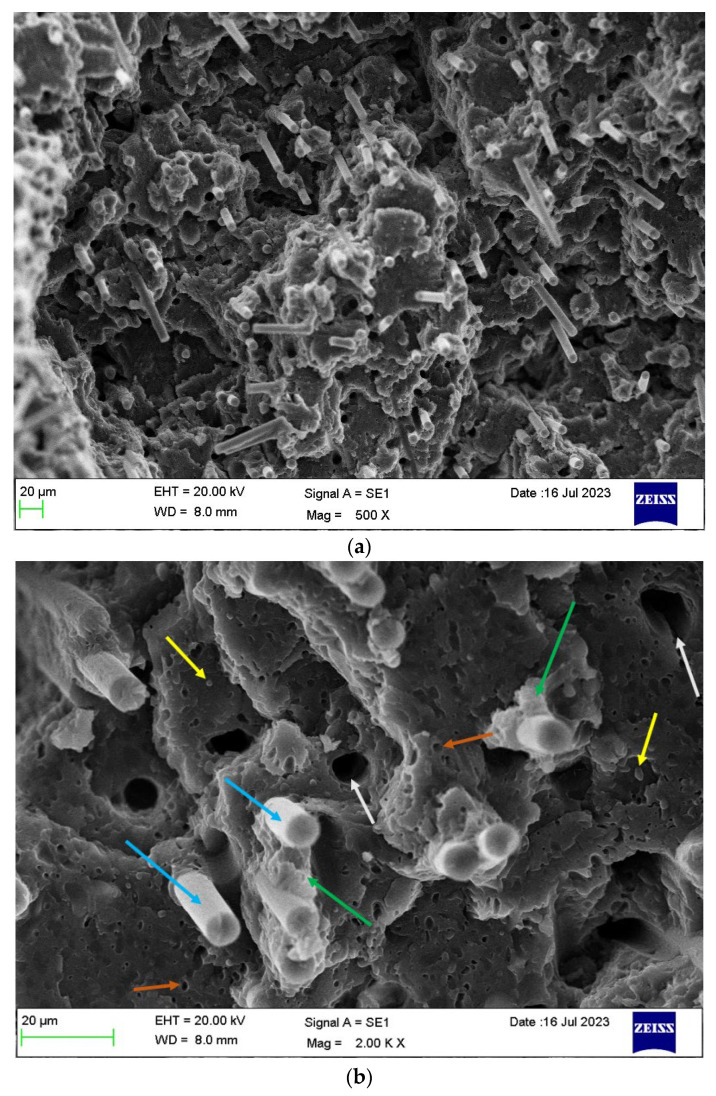
(**a**) Cryo-fractured cross section surface (Face 3’) of a bar of PBT/rPET (50/50)/Kraton (80/20) blend, with 20% CF. (**b**) Higher magnification of the same section. Well bonded fibres can be seen (the CF has broken rather than pulled out), but some have pulled out (larger circular voids). The Kraton particles are finer. Coloured arrows show the following: blue arrow show CF, white arrows show the drop outs due to CF pull out, yellow arrows show embedded Kraton particles, and the orange arrows show the drop out of Kraton and the green arrows show the good adhesion between polymer matrix and CF.

**Figure 15:**
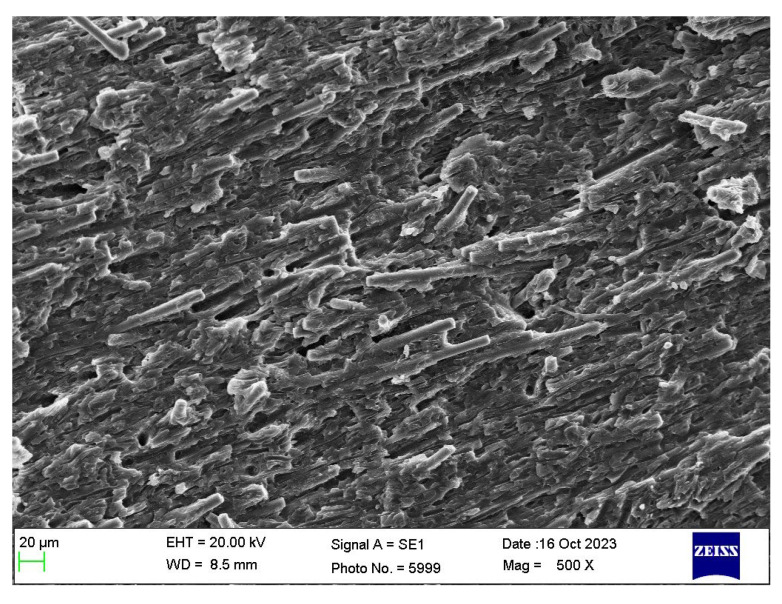
Cryo-fractured cross section surface (parallel to Face 2) of bar of PBT-rPET-Kraton−25% CF.

**Figure 16:**
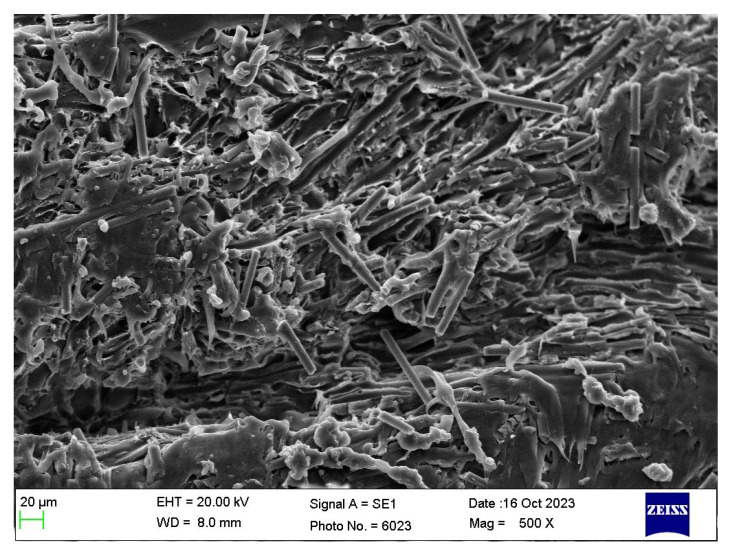
Interior of cryo-fractured surface (parallel to Face 1) of the bar of PBT-rPET-Kraton with 25% CF.

Figure 11 shows the surfaces of the bar that were imaged. The SEM images in Figure 12, Figure 13 and Figure 14 show the cryo-fractured cross section Face 3’ of the bar parallel to Face 3. Figure 15 shows the cryo-fractured cross section Face 2’ of the bar parallel to Face 2. Figure 16 shows the cryo-fractured cross section Face 1’of the bar parallel to Face 1.

Figure 12a,b show the cryogenic fracture morphology of the PBT/rPET (50/50)/Kraton (80/20) blend, without the CF. The Kraton is seen as droplets in the polyester blend, with domain size of ~1.5 μm and interparticle distance of about the same. Since Kraton is a SEBS block copolymer, the actual rubbery component (ethylene butadiene) will be smaller than 1.5 μm.

Figure 12 indicates a large number of adhered Kraton particles but also holes due to drop outs of the Kraton particles. Dispersed rubbers, below their T_g_, do not toughen plastics effectively as glassy particles are unable to deform, and thus debonding occurs when stress is applied. Figure 12b shows a higher magnification of the cryo-fracture surface, indicating Kraton particles that are well embedded as well as voids. Figure 10 showed a large increase in impact resistance with 20% Kraton, hence on balance, the Kraton would have been well adhered and effective at room temperature. Cryo-fracturing is a standard SEM preparation technique. On fracturing at cryogenic temperatures, the rubber particles would shrink (the T_g_ of Kraton rubber is −50 °C) and become brittle. Hence, the large number of dropouts in Figure 12 are the result of the sample preparation method and is not representative of what happens at room temperature.

Figure 13a shows the cross-sectional morphology of the 5% CF + 95% ((PBT/rPET, 50/50)/Kraton, 80/20). At this low loading of CF, there is not much flow orientation effect as the CF lie at various angles. Figure 13b shows a higher magnification picture. The CF is 6 μm in diameter and this particular fibre shows debonding on one side, but this was rare. The 1.5 μm Kraton particles can be seen, some embedded, and some voids are left from the drop outs.

Figure 14a shows the cryo-fracture surface of a bar of PBT/rPET (50/50)/Kraton (80/20) blend, with 20% CF. As the fibre concentration increases, more fibres become parallel, that is appear normal to the fracture surface. The higher magnification picture in Figure 14b confirms the fibres are well embedded in the matrix and there is a coating of the matrix on broken fibres.

Figure 15 shows the cryo-fractured cross section Face 2’ parallel to Face 2 of a bar of the PBT/rPET (50/50)/Kraton (80/20) blend, with 25% CF. As the fibre concentration increases, more fibres become parallel to the flow.

Figure 16 shows the cryo-fractured cross section Face 1’ parallel to Face 1 of the bar of PBT/rPET (50/50)/Kraton (80/20) blend, with 25% CF. In the interior of the bar, the fibres are more randomly oriented. The literature shows mould filling involves fountain flow, with orientation parallel to the flow and random orientation in the core [28]. The random orientation is in fact better for the electrical conductivity as Figure 16 shows some fibres are touching.

## 4. Conclusions

In this work, we have attempted to make a conductive plastic that retains good mechanical properties. A polyester blend of 50/50 PBT/rPET was selected as the matrix, not so much because it forms a co-continuous morphology, but because it is an injection mouldable composition with low viscosity, where both components crystallise in the time scale of the injection moulding.

Chopped carbon fibres (6 mm) were selected as the conductive filler. The method of debundling the carbon fibres was important to obtain good mechanical properties. An optimum mechanical method was found that retained the size on the fibre, while disaggregating the fibres without losing the ability to feed them, and without too much damage to the fibre length. The electrical conductivity showed that percolation occurs at about 10% of CF. At 20–30% CF, the electrical conductivity reached 3.414 S/m with 20% CF, 13.760 S/m with 25% CF and 40.933 J/m with 30% CF. That is, they reached the range for the EMI application, while the values for 10 and 20% CF were well above that needed for ESD. There was only a modest increase in thermal conductivity.

The mechanical properties needed to be secured. The 50/50 PBT/rPET blend was brittle with a very low notched Izod impact resistance of 11.4 J/m. Hence, it was toughened with 20% SEBS rubber. The impact resistance increased thereby to 83 J/m. As expected, addition of the 20% rubber came with a decrease in modulus and strength. Addition of the CF however compensated for the decrease in modulus and strength. The impact resistance with the 20% CF was 63.1 J/m.

SEM pictures showed the bonding of the CF and rubber particles to the matrix (polyester blend) was generally good, although the cryo-fracture method of sample preparation led to some drop outs of the Kraton particles.

The toughened bars had a high shrinkage resistance and can be used at 170 °C. The bars did not lose shape even at 230 °C, which is above the melting point of PBT, at least for short times (1 h). Of course, the conductive article with CF could only be one shade: black.

## Figures and Tables

**Table 1:** Composition details of the composite specimens.

S. No.	Sample ID	Composition (wt.%)
1	Neat Blend (50:50 PBT:rPET)	50 wt.% PBT and 50 wt.% rPET
2	Kraton SEBS Rubber	33.33 wt.% Kraton FG1901 GT and 66.67 wt.% Kraton G1657 VS
3	Kraton SEBS Rubber-Blend (KRB)	20 wt.% Kraton and 80 wt.% Neat Blend
4	5CF-KRB	5 wt.% CF and 95 wt.% KR-Blend
5	10CF-KRB	10 wt.% CF and 90 wt.% KR-Blend
6	15CF-KRB	15 wt.% CF and 85 wt.% KR-Blend
7	20CF-KRB	20 wt.% CF and 80 wt.% KR-Blend
8	25CF-KRB	25 wt.% CF and 75 wt.% KR-Blend
9	30CF-KRB	30 wt.% CF and 70 wt.% KR-Blend

**Table 2:** Change the length of the moulded bars after annealing at 170 °C for 1 h.

Composite	Initial Length (mm)	Final Length (mm)	Difference (mm)	Shrinkage %
Polyester Blend without Kraton	60	58.95	1.06	1.77
Polyester Blend with Kraton	60	59.24	0.76	1.27
5% CF	60	59.95	0.05	0.08
10% CF	60	60	0	0
15% CF	60	60	0	0
20% CF	60	60	0	0
25% CF	60	60	0	0
30% CF	60	60	0	0

## Data Availability

Data are available within the article.
